# Adenovirus—Extracellular Protein Interactions and Their Impact on Innate Immune Responses by Human Mononuclear Phagocytes

**DOI:** 10.3390/v12121351

**Published:** 2020-11-26

**Authors:** Coraline Chéneau, Eric J. Kremer

**Affiliations:** Institut de Génétique Moléculaire de Montpellier, Université de Montpellier, CNRS (French National Centre for Scientific Research), 34090 Montpellier, France; coraline.cheneau@igmm.cnrs.fr

**Keywords:** adenovirus, innate immunity, antibody, antimicrobial peptide, coagulation factor, complement, dendritic cell, host defense peptide, alarmin

## Abstract

The aim of this review is to highlight how, in a syngeneic system, human mononuclear phagocytes respond to environments containing human adenovirus (HAdV) and soluble extracellular proteins that influence their innate immune response. Soluble extracellular proteins, including immunoglobulins, blood clotting factors, proteins of the complement system, and/or antimicrobial peptides (AMPs) can exert direct effects by binding to a virus capsid that modifies interactions with pattern recognition receptors and downstream signaling. In addition, the presence, generation, or secretion of extracellular proteins can indirectly influence the response to HAdVs via the activation and recruitment of cells at the site of infection.

In this review, we highlight how human mononuclear phagocytes respond to a milieu containing human adenoviruses (HAdVs) or HAdV-based vectors and an array of soluble extracellular proteins. The response by phagocytes, which include monocytes, macrophages, conventional and plasmacytoid dendritic cells (cDCs and pDCs, respectively), orchestrates long-term immune responses. *Adenoviridae* is a family of ~150 mDa, 90 nm diameter, nonenveloped, proteinaceous particles containing a linear double-stranded DNA genome of 26–48 kb [1]. Adenoviruses (AdVs) infect mammals, reptiles, birds, fish, and amphibians. Likely due to their impact on human health, the majority of the ~200 officially recognized AdVs has been isolated from humans [2]. In most cases, human AdVs cause self-limiting respiratory, ocular, or gastro-intestinal tract infections in all populations. HAdVs are currently classified into seven species (human adenovirus A to human adenovirus G, HAdV-A to HAdV-G) and ~100 types (identified by serology and/or sequence phylogeny) [1]. Over the last 50 years the vectorization and immunogenicity of HAdVs have been areas of significant interest in the context of vaccines, oncolytics, gene transfer vectors, and morbidity in immune-compromised individuals [3,4,5]. HAdV-based vaccines, including live virus or replication-defective vectors, are used to prevent acute respiratory disease in military recruits [6] (e.g., HAdV type 4 (HAdV-E4) and HAdV-B7), or in preclinical and clinical trials targeting Malaria, Ebola virus, and SARS-CoV-2 [7,8,9,10,11,12].

Contrary to conventional wisdom, very little clinical data exist that demonstrate that HAdVs stimulate the maturation of human mononuclear phagocytes [13]. This is also the case in vitro unless high doses (100,000–200,000 particles/cell) are used [14]. Many environments exist where encounters between HAdVs and mononuclear phagocytes differ, which will differentially orient a de novo adaptive or memory response. In the case of natural infection, HAdVs will first encounter mucosa (respiratory, gastrointestinal, or ocular) surfaces. Whereas, in the case of vaccines, oncolytics, and gene therapy, HAdV vectors could be delivered intradermal, intramuscular, intravenous, orally, to the respiratory tract, in the brain, or directly into tumors [15]. Variations in frequency and activity of immune cell populations present in, or migrating to, different sites of delivery lead to differences in responses. In addition, a fourth dimension—time—needs to be incorporated into the dynamics of how a host responds to these multiparametric encounters.

## 1. Extracellular Environments

Soluble extracellular proteins englobe a spectrum of compounds that are secreted from cells in their mature form or modified by extracellular processing [16]. In plasma, albumin (54%), globulins (37%) and fibrinogen (7%) represent the major constituents [17]. Antibodies (Abs) are the first globulins to come to mind when considering a response to viruses. Although coagulation factors, complement components and antimicrobial peptides (or proteins) (AMPs) make up a small proportion of serum components, their impact on innate and adaptive immune responses is considerable. AMPs are secreted by epithelial cells and/or immune cells; Abs can be found in the systemic circulation or in the mucosa, complement components and coagulation factors are in the plasma [18].

## 2. Immunoglobulins

In the context of interactions with HAdVs, IgMs (naturally occurring, typically pentameric, high avidity and the first immunoglobulin to encounter pathogens), IgGs (the most prevalent antigen-specific immunoglobulins induced rapidly after antigen encounters), and IgAs (found preferentially in mucosal tissues) are most relevant. Numerous studies have demonstrated that antibody–antigen complexes are efficient stimulators of DC maturation and inflammation [19]. However, the scenario of intravenous injection of large doses of HAdV vectors in an immunologically naïve patient is a situation that would be rarely encountered (in contrast to assays in mice). More than 90% of the world population have encountered at least one, and likely several HAdVs types, before reaching the age of 5 years old [20]. Therefore, it is not surprising that HAdV-ICs (immune complexes) are found in some individuals with severe HAdV disease [20,21]. An immunology paradigm, based on the ability of some Abs to neutralize infection of some cells, predicts that Abs protect us from pathogens. By contrast, antibody-dependent enhanced infection can make some viral encounters worse for the host. A recent example of this is how some B-cell responses to flavivirus infections increase the pathogenicity of other members of this family [22,23]. We and others have repeatedly shown that while an Ab can neutralize HAdV infection of epithelial-like cells; this does not correlate with neutralization on cells expressing Fcγ receptors (FcγR) [24,25,26,27].

The mechanistic difference here lies in part in receptor use and intracellular processing: immunoglobulin neutralization of HAdVs by epithelial cells occurs primarily by inhibiting interactions with the virus receptor, or by post-internalization mechanisms that prevent capsid disassembly and degradation by lysosomes. At least in the case of HAdV-C5, the primary receptor switches from coxsackievirus adenovirus receptor (CAR) for epithelial cells to DC-SIGN (dendritic cell-specific intercellular adhesion molecule-3-grabbing non-integrin also is a C-type lectin receptor) for DCs and macrophages [28] (this is unlikely the case for HAdVs that use CD46 (some HAdV-Bs) or sialic acid-modified proteins motifs (some HAdV-Ds)). We are unaware of studies that map the region of HAdV-C5 that interacts with DC-SIGN and whether naturally-occurring Abs block this interaction.

Once in the cytoplasm, HAdVs encounter a vastly different environment in epithelial cells versus phagocytes. Notably, DCs slowly degrade proteins to allow cross-presentation via the MHC I complex [29]. Therefore, whether a HAdV is taken up by direct interaction with a receptor, or by redirecting to FcγRs expressed by phagocytes, dictates how the immune response develops. Differential internalization pathways are supported by the observation that HAdV-IC are found clustered into large aggregates in DCs [24], which may facilitate the escape from lysosome-mediated degradation by some particles. Perreau et al. showed that HAdV-IC s bind FcγRs and are internalized by human DCs [13]. Once HAdV-ICs access the cytosol they may also be engaged by tripartite motif-containing protein 21 (TRIM21) via the Fc portion of the Abs [30]. TRIM21 may contribute to the efficient cytosolic neutralization of mAb-bound HAdV-C5 in vitro [31] and in vivo (in mice) [32]. Again, in a cellular model, Ewan et al. showed that the more TRIM21 is expressed, the more the virus is neutralized [33]. Eichholz et al. then extended these observations to show that HAdV-ICs induce absent in melanoma 2 (AIM2) engagement and pyroptotic DC death. Pyroptosis, an inflammatory cell death that occurs in some mononuclear phagocytes following activation of inflammasome formation, leads to the release of pro-inflammatory cytokines such as TNF, IL-6 and IL-1β [24]. Under natural HAdV infection conditions, this may be a pro-host response. However, in the context of using a HAdV-C5 vector as a vaccine against HIV infections [34,35], pyroptosis may have set up a dynamic situation where HAdV-specific T cells were induced to proliferate, home to mucosal sites, and become prime targets for HIV infection [36,37].

Although the analyses are low in power and irreproducible due to sample availability, antibody-dependent enhanced infection of mononuclear phagocytes might have been involved in the death of a patient going under HAdV-C5-mediated gene therapy for ornithine transcarbamylase deficiency in the 1990’s [38]. These data challenge the safety of HAdV-based therapy in patients with preexisting anti-HAdV immunity [12]. The impact of anti-HAdV Abs also took another twist when Tran et al. [27] showed that the environment created by DCs undergoing HAdV-IC-induced pyroptosis induces bystander DCs to become tolerogenic. These antigen-loaded tolerogenic DCs can drive naïve T cells to mature into HAdV-specific T_REGs_ [27]. What role HAdV-specific T_REGs_ play in HAdV infection and disease is an open question because their effect can be extremely plastic. We have hypothesized that they contribute to persistent HAdV infections and the reduction of possible immune-mediated tissue damage. Another possibility is that harboring a robust anti-HAdV response may generate cross-reactive B-cell responses to more virulent pathogens [39].

## 3. Complement

The complement system is a network of more than 50 soluble and cell-bound molecules, produced by essentially all cells in mammals, that contribute to innate and adaptive immunity [40]. This self-amplifying cascade of messenger and effector molecules detects breaches in tissue homeostasis and pathogens. There are three canonical complement pathways: classical (predominantly Ig-mediated); alternative (interaction of complement proteins with microbial components); and mannose-binding lectin [41]. A noncanonical pathway consists of an Ig-dependent, component 3 (C3)-independent mechanism [42]. Typical complement-induced responses are self-limiting, but uncontrolled amplification contributes to disseminated intravascular coagulation, sepsis, and adult respiratory distress syndrome [43].

The classical and alternative pathways are the most relevant in the context of HAdVs. In the case of the classical pathway, the attachment of Ig to a HAdV capsid induces binding of C1q to initiate the cascade (numerous reviews can be found describing the C1q–C9 cascade and therefore it will not be repeated here [44]). In pioneering studies, Cichon et al. showed that HAdV–C5-ICs can activate complement [45]. Interestingly, they also were the first to show that high concentrations of HAdV–C5 directly activate a complement pathway, but did not characterize the process. Later, it was shown that HAdV–C5 can induce the alternative complement pathway by binding C3 directly [46]. Lastly, a C1q-dependent mechanism can neutralize HAdV–C5 by covalently linking C4b, which then targets the complex for gC1qBP-mediated uptake where HAdV–C5 is degraded in lysosomes [47]. It is also worth noting that there are exceptions: although human sera can contain Abs that bind canine adenovirus type 2 (CAV-2), the classical and alternative complement pathways are not engaged due to a block upstream of the C3 convertase and interplay between the C1q–C1r2–C1s2 complex and C1q inhibitor and CAV-2 [48].

As the focus of this review is to link HAdVs, extracellular proteins, and human phagocytes, we have focused on the cellular events downstream of complement activation. All HAdV-associated complement pathways result in the generation of the anaphylatoxins C3a and C5a. Via their cognate receptors C3aR, and C5aR1, and C5aR2, C3a, and C5a exert regulatory effects on the humoral and cellular arms of innate immunity [49]. For example, C3b induces IL-1 and TNF release [50] and activation of phagocytes [51]. Yet, unexpectedly, using an in vitro model system, Zaiss et al. concluded that complement did not affect HAdV-C5 entry or activation of human macrophages [25]. While possible in mice, it is unknown if HAdV-C5 uptake by human neutrophil is affected by the presence of complement or Abs. Notably, HAdV-C5 and neutrophil interactions were significantly reduced when they were incubated in IgG-depleted or serum containing complement [52].

C5a is a potent inflammatory peptide with a broad spectrum of functions including acting as a chemoattractant for neutrophils, monocytes, and macrophages [53]. In humans, C5aR1 is on the surface of most DC subsets, including monocyte-derived DCs, dermal DCs, and Langerhans cells [54]. The direct effects of C5aR1 engagement leads to the activation of pattern-recognition receptors and FcγRs. Examples of this cross-talk include C3a/C3aR-mediated activation of the inflammasome in human macrophages, monocytes and DCs [55,56], and C5a-mediated suppression of Toll-like receptor (TLR)-induced IL-12 production from macrophages [57], as well as C5a-mediated activation of phagocytes through IC engagement of the inhibitory IgG-binding FcγRIIb with Dectin-1 [58]. C3a and C5a are also involved in the development of Th2 and Th17 immunity through the tweaking of DC activation [59], and the induction of regulatory T cells (T_REGs_) [60]. Whether complement-linked induction of T_REGs_ synergizes with the HAdV-IC induced tolerogenic DC and T_REGs_ [27] is unknown and worthy of more investigation.

On the flip side, thrombocytopenia and anemia can be associated with some severe cases of adenoviremia. Why is this relevant to complement activation? In a GPIb- and C3-dependent manner, platelets can deliver pathogens to tissue resident DCs and increase the pathogen directed immune response [61]. As mentioned above, the direct binding of C3 to some HAdV capsids could influence the anti-HAdV response when they are found in the systemic circulation (whether due to wild-type infections or delivery for gene therapy). In addition to platelets, some adenovirus types that bind CAR induce hemagglutination of human erythrocytes [62]. Therefore, immune adherence may also contribute to the complement-associated response to HAdVs [63] as C3–HAdV complexes could be delivered to the complement receptor 1 (CR1) clustered on the surface of human erythrocytes [64]. It is worth noting that C5a can also activate the coagulation pathway [65], which brings immunoglobulins, complement, and coagulation factors into the global discussion of an anti-HAdV immune response.

## 4. Antimicrobial Peptides (AMPs)

AMPs are effector molecules of the innate immune system and can act as an endogenous antimicrobial against a broad array of infectious agents [66,67,68,69]. More than 1300 AMPs have been identified and many are produced by epithelial cells of skin, oral mucosa, and gastrointestinal tract, and by myeloid cells, including neutrophils, which are among the first leukocytes to infiltrate infected and vector-injected tissues [36]. The ubiquitous expression and rapid production of AMPs allow them to act as first line responders to pathogens [69,70]. In addition to responses to pathogens, AMPs can recruit and modulate the activities of neutrophils, monocytes, macrophages, DCs and T cells. AMPs can induce the expression of cytokines, neutralize endotoxin and induce angiogenesis [66,67,68]. Minor variations in the primary sequence of an AMP can induce major changes in their activity and, therefore, syngeneic systems are the gold standard to understand clinically relevant scenarios [68]. Notably, pockets of negatively charged areas in the hexon hypervariable regions of many HAdV types [71,72,73,74,75] favor binding of positively charged AMPs.

### 4.1. Alarmins

Among the AMPs is a subset dubbed “alarmins”, which include defensins, lactoferrin (Lf), and LL-37. Defensins are ~30 amino acid cationic peptides with a conserved framework of six disulfide-linked cysteines. Two α-defensins, human neutrophil peptides 1 and 2 (HNP-1 and -2) stimulate monocyte, DC and T cell recruitment [76,77,78,79]. The four human β-defensins (hBD1–4) stimulate recruitment of monocytes, DCs, T cells and mast cells [80,81,82]. Both α- and β-defensins also have selective actions: α-defensins induce the recruitment of human CD8^+^ and CD4^+^/CD45RA^+^ naïve T cells, whereas β-defensins induce recruitment of immature DCs and CD4^+^/CD45RO^+^ memory T cells [79]. Furthermore, during interaction with TLR-1 and -2 on monocytes and DCs, hBD3 can induce upregulation of costimulatory molecules CD80, CD86 and CD40 [83].

Lf, an atypically large alarmin (80 kDa), is a multifunctional member of the transferrin family that sequesters iron and is found in various secretory fluids, such as milk, saliva, tears, and nasal secretions. Lactoferrin induces DC maturation via DC-SIGN or targeting TLR-2 and -4 and drives Th1 responses [84,85,86,87]. Lf also induces DC maturation. Lactoferricin, a biologically active N-terminal fragment of 49 aa, also exhibits immunomodulatory effects, such as stimulation of monocyte differentiation and release of pro-inflammatory cytokines [88].

LL-37 is a 37 amino acid cationic peptide found in lysosomes of macrophages, polymorphonuclear leukocytes, and keratinocytes. LL-37 serves a critical role against infections by its chemotactic activity [89], influencing phagocytosis [90], TLR activation [91,92,93], DC differentiation, and induced T-cell polarization [94].

### 4.2. How Alarmins Influence HAdVs

Alarmins influence HAdV infections and immunogenicity via mechanisms that vary in each model system. HNP-1 and human defensin 5 (HD5) impair HAdV-C5, -D12 and -B35 infection of epithelial cells by stabilizing an intrinsically disordered region of the vertex and thus prevent initial stages of viral disassembly post-entry [95,96,97,98]. By contrast, based on protein separation using bronchoalveolar lavage, Skrygan et al. concluded that defensins are not important anti-HAdV-C5 factors during infection of lung epithelium [99]. Recently, Tartaglia et al. showed that HD5 regulates immunogenicity by increasing HAdV-D26 and HAdV-D48 infections in mice (but not HAdV-C5 and HAdV-B35) [100]. It is relevant to note that HD1, 3, and 4 are elevated in nasal aspirates from children with HAdV infections [101]. While the authors assumed that increased levels of defensins should be pro-host, this assumption is challenging to demonstrate in a clinical setting. While not directly related to human phagocytes, tumor-derived HD5 affected oncolytic HAdV-B3 replication during cancer therapy [102]. Interestingly, HD5 inhibition was circumvented by the capacity of HAdV-B3 to produce virus-like particles made up of penton-dodecahedra that sequester and neutralize HD5.

Arnberg and colleagues demonstrated that lactoferrin binds to the negatively-charged hexon hypervariable regions of HAdV-C5, -A31 and -B35 [75], thereby acting as a bridge to increase infection in human epithelial-like cells [103]. Notably, enhanced infection is independent of CAR [104], the primary cellular attachment molecule for HAdV-C5 and -A31 [105]. Lf also mediates increased HAdV-C5 transduction in human monocytes, pDCs, cDCs, and Langerhans cells possibly via the engagement of DC-SIGN [86] and other factors. Interestingly, human Lf has no effect on HAdV-C2 infection of the above phagocytes while bovine Lf increased HAdV transduction [106].

Finally, Uchio et al. found LL-37 also inhibits propagation of HAdV-B3, -D8, -D19a, and -D37, but not -E4 (which is likely of simian origin and now endemic in some military training camps) infection of A549 cells [107]. By contrast, Gordon et al. found that LL-37 inhibits the propagation of HAdV-D19 (but no significant inhibition on HAdV-B3, -C5, or -D8) [108]. These differences may be due to the experimental design with respect to the sequential versus simultaneous addition of virus and LL-37, and the dose of LL-37.

## 5. Coagulation Factor

The coagulation system is a multiprotein cascade that is involved in blood clot formation [109]. Secreted in the systemic circulation as inactive zymogens, the vitamin K-dependent coagulation factors VII, IX and X all share a common structural N terminal γ-carboxyl glutamic acid domain and a C terminal serine protease domain. It is the N terminus that binds with nanomolar affinity to some of the hexon hypervariable regions [110]. Based on HAdV pathogenicity, it is difficult to explain what selective advantage binding to vitamin K-dependent coagulation factors adds. Numerous studies and reviews address the impact of vitamin K-dependent coagulation factors on HAdV-mediated gene transfer in mice [111,112,113,114], with the global conclusion that vitamin K-dependent coagulation factors act as bridges between the negatively-charged capsid and negatively-charged heparan sulfate proteoglycans. The use of coagulation factors by HAdV may be important for liver tropism following intravenous injection of HAdV-C5 vectors, and during naturally occurring infections. Indeed HAdV-C5 and -A31 require FIX or FX to infect human corneal epithelial (HCE) cells and human lung carcinoma (A549) cells [115]. It is noteworthy that Eichholz et al. found that human FX coupled to HAdV-C5 did not induce greater uptake by, or maturation of, human DCs [116]. Therefore, the mechanism by which some coagulations factors modify the tropism and response to some HAdVs and their vectors needs greater species-specific analyses. That being said, Alba et al. quantified HAdV-C5 and FX-binding-ablated HAdV-C5-mediated gene transfer in a non-human primate and found that HAdV-C5 vectors efficiently mediated gene transfer to the liver, whereas FX-binding-ablated vectors primarily targeted the spleen. Therefore, some data generated using mice may be consistent with the mechanism underlying hepatocyte transduction in humans [117].

## 6. Perspectives

Whenever possible, we focused on how HAdVs and extracellular proteins impact the innate immune response in human phagocytes. A caveat though is that the use of replication-defective HAdV vectors precludes the analyses of the impact of extracellular proteins on events downstream of entry into the nucleus. When necessary we have introduced data generated in other systems (e.g., rodents) to provide background. For example, HAdVs alone readily induce the maturation of murine DCs, but poorly induce human DC maturation [13,118]. We know that coagulation factor-specific interactions influence HAdV innate immune responses differentially in mouse and in human phagocytes. Murine FX-armored HAdVs stimulated murine macrophages in vitro and in vivo, whereas human FX–HAdV complexes had no effect on human DCs in vitro [116,119]. While bovine Lf increases HAdV-C2 infection of human DCs, human Lf has no effect [106]. Nonetheless, data from rodents can generate avenues to explore in humans. For example, Gounder et al. found that murine α-defensins protect mice from oral infection by mouse adenovirus 1 (MAdV-1). Yet, in spite of the sensitivity of MAdV-1 infection to α-defensin neutralization in cell culture, α-defensin production by Paneth cell had no effect on the kinetics and magnitude of MAdV-1 dissemination to the brain. This may have been due to a delay in the production of neutralizing Abs. Thus, in mice, α-defensins play a role in antiviral immunity that is distinct from their antiviral activity observed in cell culture [120].

The law of parsimony suggests synergistic interactions between Abs, coagulation factors, complement and AMPs in most pathological or vaccine-associated paradigms. Yet, some AMPs can inhibit the complement pathway. Lf, by blocking the formation of C3 convertase [121], and HNP-1, by blocking the C1q hemolytic activity [122], can inhibit the classical complement pathway. Xu et al. demonstrated that FX shields HAdV-C5 and blocks HAdV-dependent complement activation [123]. In each scenario, two parameters are crucial: site and timeline of infection. If HAdVs enter via intranasal, oral, gastrointestinal, ocular, intravenous, intradermal, or muscular tissues, the first cells to be infected, and then those recruited, will differ. For example, some phagocytes (e.g., alveolar macrophages) are recruited to specific organs and not all extracellular proteins will be all present or secreted at the same level.

The timeline of infection and extracellular protein secretion are also crucial parameters. Depending on the extracellular protein first secreted, it could change HAdV interactions with its target cell. For example, if an AMP binds first, does that interaction prevent Ab binding? Do Abs outcompete AMPs because of affinity or avidity? Are there situations where they act synergistically? Unsurprisingly, assays addressing these complex interactions are challenging because of the direct effect of some extracellular proteins on phagocyte homeostasis and recruitment. Yet, these issues are critically important to understand in the scenario where AdV-based vaccines are used [12]. It is possible that some extracellular proteins could be used as adjuvants to increase vaccine efficacy. If an AMP is used as an adjuvant, could the complex increase HAdV uptake by phagocytes? or uptake by other tissue resident cells? There exist multiple modifications of HAdV capsid sequences (or coating the capsid with protective polymers) that reduce binding of extracellular proteins [124,125,126,127]. Testing these HAdV vectors as vaccines could provide valuable information on the complex interactions that alarmins could play. We know very little about what happens in the 48 h post-vaccine injection (predominantly in muscle for vaccines): what cells are recruited and in what ratio? What do they secrete in a host with or without preexisting humoral and cellular immunity? How does this combination of cells and extracellular proteins modify the innate and adaptive immune response? Clearly multiple extracellular proteins are found in the same microenvironment and therefore addressing their impact on HAdV uptake in binary analyses poorly considers HAdV or phagocyte interactions. Add on top tissue undergoing an inflammatory reaction where the extracellular matrix, which is composed of proteins, proteoglycans, and glycoproteins each with varying physical and biochemical properties, is degraded and releases peptides. Matrix metalloproteinases, ADAMTS (a disintegrin and metalloproteinase with thrombospondin motifs), and serine proteinases (plasmin and cathepsin G) are the most common extracellular matrix degrading enzymes [128]. Very little is known about how extracellular matrix degradation modifies HAdV tropism and immunogenicity. Data that may shed light onto this process might be gleaned from oncolytic vectors that express enzymes that degrade the extracellular matrix [129].
